# Cost-effectiveness of a physical activity and behaviour maintenance programme on functional mobility decline in older adults: an economic evaluation of the REACT (Retirement in Action) trial

**DOI:** 10.1016/S2468-2667(22)00030-5

**Published:** 2022-03-21

**Authors:** Tristan M Snowsill, Afroditi Stathi, Colin Green, Janet Withall, Colin J Greaves, Janice L Thompson, Gordon Taylor, Selena Gray, Heidi Johansen-Berg, James L J Bilzon, Jolanthe L de Koning, Jessica C Bollen, Sarah J Moorlock, Max J Western, Jack M Guralnik, W Jack Rejeski, Kenneth R Fox, Antonieta Medina-Lara

**Affiliations:** a University of Exeter Medical School, University of Exeter, Exeter, UK; b School of Sport, Exercise and Rehabilitation Sciences, University of Birmingham, Birmingham, UK; c Department for Health, University of Bath, Bath, UK; d Faculty of Health and Applied Sciences, University of the West of England (UWE) Bristol, Bristol, UK; e Wellcome Centre for Integrative Neuroimaging, John Radcliffe Hospital, University of Oxford, Oxford, UK; f Birmingham Clinical Trials Unit, University of Birmingham, Birmingham, UK; g Department of Epidemiology and Public Health, University of Maryland School of Medicine, Baltimore, MD, USA; h Department of Health and Exercise Science, Wake Forest University, Winston-Salem, NC, USA; i Centre for Exercise, Nutrition and Health Sciences, School for Policy Studies, University of Bristol, Bristol, UK

## Abstract

**Background:**

Mobility limitations in older populations have a substantial impact on health outcomes, quality of life, and social care costs. The Retirement in Action (REACT) randomised controlled trial assessed a 12-month community-based group physical activity and behaviour maintenance intervention to help prevent decline in physical functioning in older adults at increased risk of mobility limitation. We aimed to do an economic evaluation of the REACT trial to investigate whether the intervention is cost-effective.

**Methods:**

In this health economic evaluation, we did cost-effectiveness and cost-utility analyses of the REACT programme versus standard care on the basis of resource use, primary outcome, and health-related quality-of-life data measured in the REACT trial. We also developed a decision analytic Markov model that forecasts the mobility of recipients beyond the 24-month follow-up of the trial and translated this into future costs and potential benefit to health-related quality of life using the National Health Service and Personal Social Services perspective. Participants completed questionnaire booklets at baseline, and at 6, 12, and 24 months after randomisation, which included a resource use questionnaire and the EQ-5D-5L and 36-item short-form survey (SF-36) health-related quality-of-life instruments. The cost of delivering the intervention was estimated by identifying key resources, such as REACT session leader time, time of an individual to coordinate the programme, and venue hire. EQ-5D-5L and SF-36 responses were converted to preference-based utility values, which were used to estimate quality-adjusted life-years (QALYs) over the 24-month trial follow-up using the area-under-the-curve method. We used generalised linear models to examine the effect of the REACT programme on costs and QALYs and adjust for baseline covariates. Costs and QALYs beyond 12 months were discounted at 3·5% per year. This is a pre-planned analysis of the REACT trial; the trial itself is registered with ISRCTN (ISRCTN45627165).

**Findings:**

The 12-month REACT programme was estimated to cost £622 per recipient to deliver. The most substantial cost components are the REACT session leader time (£309 per participant), venue hire (£109), and the REACT coordinator time (£80). The base-case analysis of the trial-based economic evaluation showed that reductions in health and social care usage due to the REACT programme could offset the REACT delivery costs (£3943 in the intervention group *vs* £4043 in the control group; difference: –£103 [95% CI −£695 to £489]) with a health benefit of 0·04 QALYs (0·009–0·071; 1·354 QALYs in the intervention group *vs* 1·314 QALYs in the control group) within the 24-month timeframe of the trial.

**Interpretation:**

The REACT programme could be considered a cost-effective approach for improving the health-related quality of life of older adults at risk of mobility limitations.

**Funding:**

National Institute for Health Research Public Health Research Programme.

## Introduction

Frailty is a common consequence of ageing, resulting in individuals becoming less able to do their usual activities and take care of themselves and their loved ones, and putting them at greater risk of future injury and disability. Physical activity interventions to maintain mobility and prevent frailty have the potential to improve quality of life for older people,^1^ as well as reduce the extent to which they use health-care resources.

The Retirement in Action (REACT) programme was designed as a community-based, multimodal, group physical activity intervention focusing on improving strength, balance, and mobility in older adults at risk of impaired lower extremity function. The programme also had social and educational components designed to encourage the maintenance of healthy behaviours. The REACT trial (registered at ISRCTN, ISRCTN45627165) was a pragmatic, multicentre, two-arm, single-blind, parallel-group, randomised controlled trial (with an internal pilot phase),^2^ assessing the effectiveness of the REACT programme for community-dwelling adults aged 65 years and older with some limitations in mobility and not in full-time employment. The primary outcome of the REACT trial was the Short Physical Performance Battery (SPPB), which is a measure of lower-limb function. SPPB score has been shown to be associated with health-care resource utilisation^3^ and predictive of future major mobility-related disability^1^ and mortality risk.^4^ The REACT trial showed that SPPB was higher in the intervention group than in the control group (adjusted mean difference of 0·49 [95% CI 0·06–0·92]) 24 months after randomisation (ie, 12 months after completion of the intervention).^5^

**Research in context**

**Evidence before this study**

We did a pragmatic literature review to identify existing economic evaluations of interventions with a physical activity component among older adults, without selection for current or previous health conditions and with the aim of the intervention to maintain mobility or delay the incidence of frailty. We searched MEDLINE (via Ovid) using MeSH terms and the economic evaluation study design filter used by the National Health Service Economic Evaluations Database. We identified two relevant publications. In 2019, Alhambra-Borrás and colleagues did a model-based economic evaluation of a group-based physical exercise intervention for community-dwelling older adults. They concluded that the intervention would lead to cost savings (from a health-care perspective) and quality-adjusted life-year (QALY) gains, and therefore would be dominant, but with a risk of bias. In 2016, Groessl and colleagues did an economic evaluation alongside a randomised controlled trial of a physical activity programme (including group and home sessions) in older adults at risk of developing major mobility-related disability. They found that the physical activity programme was more costly than the comparator (health education) and led to QALY gains. Although the effect did not reach statistical significance, there were higher health-care resource utilisation costs in the physical activity group.

**Added value of this study**

This study adds robust evidence that a 12-month physical activity intervention can lead to meaningful and sustained improvements in quality of life, at a cost that is justifiable as a use of limited resources. The possibility of reduced health-care resource utilisation means the intervention is very likely to be cost-effective.

**Implications of all the available evidence**

Declining physical function with age is associated with reductions in quality of life and increasing health-care resource utilisation. A 1-year group-based physical activity intervention (with social and behaviour change elements), when targeted at older adults at risk of mobility-related disability (ie, with an Short Physical Performance Battery score between 4 and 9), can improve physical function and quality of life in a cost-effective manner.

To establish whether limited resources should be spent on interventions, it is necessary to consider whether the benefits derived outweigh the benefits forgone if spending elsewhere needs to be reduced to remain within a fixed budget. Health economic evaluations estimate the relative costs and benefits of different interventions and combine these into a measure of cost-effectiveness called the incremental cost-effectiveness ratio (ICER). The ICER is the ratio of the added costs of an intervention to the added benefits, which are typically measured using quality-adjusted life-years (QALYs)—a utility measure that combines the duration of life lived with the quality of life experienced. In the UK, a health intervention is generally considered cost-effective if the ICER is less than £20 000–30 000 per QALY gained.^6^ In this study, we aimed to conduct a health economic evaluation of the REACT programme.

## Methods

### Study design

In this health economic evaluation, we report an estimation of the cost of delivering the REACT programme, as well as cost-effectiveness analyses based on the data collected within the 24-month duration of the REACT trial (trial-based economic evaluation), and an extrapolation of costs and QALYs over the remaining lifetime of the participants using a decision analytic modelling framework. The analysis was conducted in line with a pre-specified health economic analysis plan, which is available from the authors upon request; there were no substantial deviations from this analysis plan. In addition, we did the evaluation in line with the National Institute for Health and Care Excellence (NICE) reference case^6^ and reported it in line with the Consolidated Health Economic Evaluation Reporting Standards checklist.^7^

### Interventions

Between February, 2016, and October, 2017, 777 par-ticipants with an SPPB score between 4 and 9 inclusive, recruited from three sites in England,^8^ were randomly assigned to receive either a 12-month group exercise and behavioural maintenance programme delivered in leisure or community centres by qualified and trained exercise professionals or three healthy ageing education workshops covering healthy ageing topics (eg, healthy eating, dealing with dementia, and volunteering), each lasting 60–90 min, delivered before the 6-month, 12-month, and 24-month assessments. The target group size was 15 individuals and sessions were initially twice a week, reducing to once a week after 12 weeks, for a total of 64 sessions lasting 80–105 min each. Further details about the REACT intervention are given in the study protocol.^2^

### Data collection

Clinical effectiveness was assessed using the SPPB by investigators masked to participant allocation. SPPB measures normal gait speed, chair rises, and standing balance, and has a summary score from 0 to 12, which is the sum of three scores, each from 0 (unable to complete) to 4 (best performance). Questionnaire booklets were distributed to participants, which included measures of health-related quality of life and health and social care resource use. Health-related quality of life was assessed using the EQ-5D-5L^9^ and the 36-item short-form survey (SF-36).^10^ These are both generic health-related quality-of-life instruments widely used in clinical trials. Use of health and social care resources was assessed via a bespoke resource use questionnaire that asked participants to recall community and hospital-based health and social care resource use during the previous 6 months (eg, the number of times they had consulted their general practitioner or attended a hospital outpatient appointment). Measures were collected at baseline, and at 6, 12, and 24 months after randomisation.

The cost of delivering the intervention was estimated by identifying key resources: REACT session leader time (to prepare for, travel to and from, and deliver the sessions), the time of an individual to coordinate the programme (eg, booking venues, recruiting session leaders, maintaining a waiting list, and administering payments), and venue hire. Other resources with lower impact on overall costs were also identified (eg, consumables, refreshments, and training of session leaders). Resource use was estimated from time sheets and class registers completed by REACT session leaders, as well as investigator estimates. REACT session leaders recorded the duration and attendance of sessions, as well as any time for travel and preparation.

### Data analysis

EQ-5D-5L and SF-36 responses were converted to preference-based utility values using the crosswalk to EQ-5D-3L^11^ and a value set for the six-dimension short-form survey (SF-6D).^12^ These were used to estimate QALYs over the 24-month trial follow-up using the area-under-the-curve method.^13^ QALYs calculated from EQ-5D-5L were used in the base-case analysis in accordance with a pre-specified analysis plan.

The cost of session leader time was estimated to be £29·43/h (this includes £15·67/h of oncosts and overheads), as they are likely to have qualifications consistent with National Health Service (NHS) Agenda for Change Band 4.^14^ The venue hire cost was estimated by drawing a random sample of ten populated geographical areas in England (each was a lower-layer super output area with an average population of 1500 and one was sampled for each decile of the Index of Multiple Deprivation) and, for each, locating a suitable venue for the REACT intervention, resulting in a mean price of £17·32/h (range £12–25/h).

Unit costs for NHS and Personal Social Services (PSS) resource use were estimated primarily from the Unit Costs of Health and Social Care by the PSS Research Unit^14^ and from the NHS National Cost Collection (appendix 1 p 1).^15^ Price weights were estimated from an NHS and PSS perspective—ie, costs to patients and indirect costs to society (due to lost productivity) were not included.^6^ NHS and PSS resource use was not measured during the 12–18 months from randomisation (as questionnaire booklets were not sent at 18 months and recall beyond 6 months was considered unreasonable), so resource use in this period was estimated to be the average of resource use in the periods 6–12 months and 18–24 months after randomisation.

We used generalised linear models to examine the effect of the REACT programme on costs and QALYs and adjust for baseline covariates, including stratification variables and baseline quality of life and resource use. Missing data were handled through multiple imputation using a predictive mean matching algorithm, with 50 imputation sets generated. A significance level of 0·05 was used. Costs and QALYs beyond 12 months were discounted at 3·5% per year, in line with the NICE reference case.^6^ Costs are reported in 2018–19 pounds sterling (£).

### Long-term costs and outcomes

We constructed a Markov model with health states representing the 13 possible SPPB scores (0–12), and a health state for death (appendix 1 p 2). Markov cohort simulation was selected because it is an efficient method to estimate long-term costs and outcomes while making transparent the assumptions surrounding the disease process and its effects on costs and quality of life. An annual cycle length was used. For each combination of age (for ease of computation we used ages at 5-year intervals, from 65 to 95 years) and sex, we estimated the SPPB profile after 24 months in the intervention and control groups and then simulated the future trajectory of SPPB using a statistical model fitted to data from the control group. Other than the different SPPB profiles at the start of the model (24 months after initiating the REACT programme) and the costs and QALYs estimated during the initial 24 months from trial data, the two model groups were identical.

We estimated the NHS and PSS costs associated with living a year with a particular SPPB score (appendix 1 p 3) and the health-state utility value according to SPPB score (appendix 1 p 4), so that long-term costs and QALYs could be extrapolated. These estimates were derived from control group data only, as it was considered possible that the effects of the intervention on costs and quality of life might not be entirely mediated through SPPB.

We assumed that there would be no effect on mortality—ie, life expectancy is identical and QALY differences arise only from differences in quality of life. Although some studies have shown a link between SPPB score and mortality,^4^ we are not aware of any studies that have yet shown that an intervention generating an improvement in SPPB versus control conditions also leads to an improvement in mortality, which has been suggested as a suitable requirement for a measure to become a surrogate for mortality.^16^

In the base case, we assumed a woman aged 75 years would be receiving the REACT programme or control treatment, because 75 years was close to the mean age in the REACT study (77 years) and most participants (66%) were women.

Appendix 2 provides details for all model parameters, including ranges for one-way sensitivity analyses and distributions for probabilistic sensitivity analysis.

### Role of the funding source

The funder approved the study design but had no role in data collection, data analysis, data interpretation, the writing of the manuscript, or the decision to submit the manuscript for publication.

## Results

The REACT programme is estimated to cost £9466 per group or £622 per participant, on the basis of an average group size of 15·2 participants. The most substantial cost components are the REACT session leader time (£309 per participant), venue hire (£109), and the REACT coordinator time (£80; table 1). The intervention cost is sensitive to the cost per h and the time input of the REACT session leader, group size, and the venue cost.

**Table 1:** Cost of delivering the REACT intervention

		**Resource use per group**	**Unit cost**	**Cost per group**	Cost per participant*
Link worker		30 min per participant	£33·83/h	£257·48	£16·92
Coordinator		36 h per group	£33·83/h	£1217·88	£80·01
Introductory sessions		45 min per participant	£29·43/h	£335·99	£22·07
Equipment					
	Pedometers	One per participant	£9·95 per item	£151·46	£9·95
	Other	Ankle weights and therabands (assumes reuse and sharing between groups)	£29·16†	£29·16	£1·92
REACT session leader					
	Preparation	30 min per session	£29·43/h	£941·76	£61·87
	Travel time	30 min per session	£29·43/h	£941·76	£61·87
	Delivery	90 min per session	£29·43/h	£2825·28	£185·60
Consumables					
	Refreshments	About one per participant per session	£1·00 per item	£621·32	£40·82
	Printed materials	One set per participant	£2·00 per item	£30·44	£2·00
Venue hire		90 min per session	£17·32/h	£1662·72	£109·23
Training session leaders					
	Training leaders	One leader to four trainees	£33·83/h	£22·20	£1·46
	REACT session leaders time	1·5 days for every four programmes delivered	£29·43/h	£77·25	£5·08
Training venue		As above	£29·43/h‡	£77·25	£5·07
	Training manual	One per REACT trainer	£12·00 per item	£3·00	£0·20
Total				£9465·69	£621·83

* Based on an average group size of 15·2 participants.

† Estimated as £136·27 for equipment, used five times over 5 years and amortising with a discount rate of 3·5%.

‡ Estimated as £309 for 1·5 days based on recorded costs of £259 and £359 for training venues within the study.

Data on baseline NHS and PSS resource use (recalling 6 months before joining the study) and health-related quality of life was nearly complete, with 734 (94%) of 777 participants providing data on baseline resource use and 684 (88%) providing baseline EQ-5D-5L and SF-36 data. There were no clear imbalances in health-related quality of life between the groups, but mean NHS and PSS resource use was higher at baseline in the intervention group than in the control group (£797 *vs* £653; appendix 1 pp 5–6), primarily due to increased hospital overnight stays.

There was a moderate amount of missing data at subsequent timepoints, with 451 (58%) of 777 participants providing complete data to calculate NHS and PSS resource use and QALYs. Among available cases, the raw mean costs of primary care resource use over the 24-month trial period were similar in both groups (£820 in the intervention group *vs* £838 in the control group). Raw mean costs for hospital overnight stays were somewhat higher in the intervention group (£1105 *vs* £1032), but costs for other hospital use were substantially higher in the control group (£1373 *vs* £1856).

**Figure 1:**
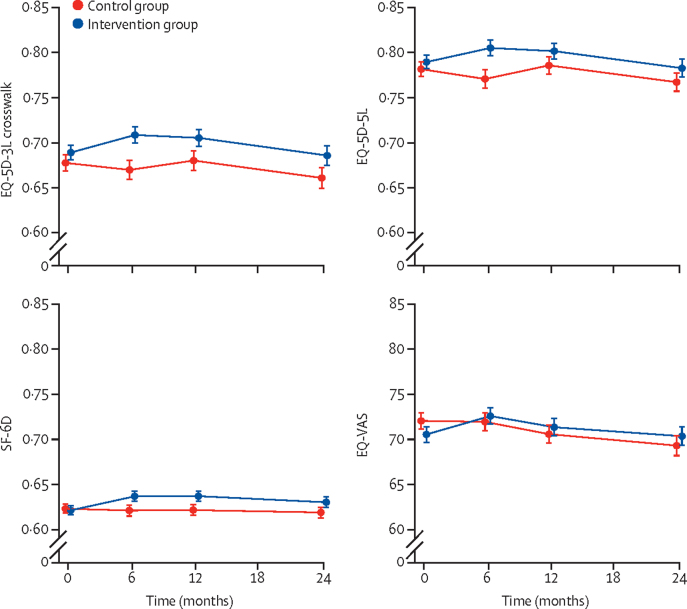
Health-related quality of life in the REACT study Error bars show ± 1 SE of the mean. The theoretical maximum (minimum) values are 1 (−0·594) for EQ-5D-3L crosswalk, 1 (−0·285) for EQ-5D-5L, 1 (0·203) for SF-6D, and 100 (0) for EQ-VAS. SF-6D=six-dimension short-form survey. VAS=visual analogue scale.

EQ-5D-5L data show that there was an increase in the proportion of participants reporting no problems with mobility (level 1) in the intervention group compared to the control group (appendix 1 pp 7–8). This and other changes meant that preference-based utility values were higher in the intervention group than in the control group (figure 1).

**Figure 2:**
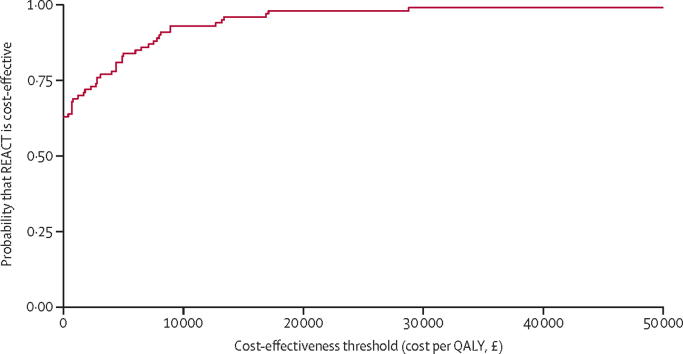
Cost-effectiveness acceptability curve from trial-based economic evaluation QALY=quality-adjusted life-year.

After multiple imputation sets were generated and regression models were fitted, it was estimated that the REACT programme would result in (non-significant) overall cost savings of £103 (95% CI −£489 to £695) and a significant gain of 0·040 (95% CI 0·009 to 0·071) QALYs. Bootstrapping was performed to produce a cost-effectiveness acceptability curve (figure 2). The upper limit of the CI for the ICER was estimated to be £17 000 per QALY using the bootstrap percentile method, suggesting that the REACT programme is very likely to be cost-effective at the standard cost-effectiveness threshold range of £20 000–30 000 per QALY (table 2).

**Table 2:** Cost-effectiveness results from trial data

	**Costs from health and social care resource use**	**Intervention costs**	**Total costs**	**QALYs**	**ICER**
**Base case (multiple imputation)**					
Intervention group	£3321	£622	£3943	1·354	..
Control group	£4046	0	£4046	1·314	..
Difference (95% CI)	−£725 (−£1316 to −£133)	£622	−£103 (−£695 to £489)	0·040 (0·009 to 0·071)	Dominant
**Complete case**					
Intervention group	£3249	£622	£3871	1·372	..
Control group	£3573	0	£3573	1·323	..
Difference (95% CI)	−£325 (−£1043 to £394)	£622	£297 (−£421 to £1016)	0·049 (0·010 to 0·089)	£6000 per QALY gained
**SF-6D for QALYs (multiple imputation)**					
Intervention group	£3321	£622	£3943	1·241	..
Control group	£4046	0	£4046	1·216	..
Difference (95% CI)	−£725 (−£1316 to −£133)	£622	−£103 (−£695 to £489)	0·025 (0·006 to 0·044)	Dominant

ICER=incremental cost-effectiveness ratio. QALYs=quality-adjusted life-year. SF-6D=six-dimension short-form survey.

A number of sensitivity analyses were done to explore the effect of assumptions on cost-effectiveness. When NHS and PSS resource use impact was assumed to be zero, the ICER was estimated to be £15 650 per QALY. When the SF-6D was used to calculate utilities, the ICER was estimated to be £4150 per QALY. When these two assumptions (no resource use impact and SF-6D utilities) were combined, the ICER was estimated to be £25 050 per QALY. When complete-case analysis was used instead of multiple imputation, the REACT programme was estimated to lead to (non-significant) additional costs of £297 (95% CI −£421 to £1016) and 0·049 additional QALYs (0·010 to 0·089). This analysis resulted in an estimated ICER of £6000 per QALY. The sensitivity of incremental costs to the unit costs of health and social care resources was assessed using multiple one-way sensitivity analyses (appendix 1 p 9)—these showed that, although there was some sensitivity to the unit costs of some hospital resources, the difference this could make (±£40) is small in comparison to the central estimate for incremental costs.

In the base-case analysis (assuming a woman aged 75 years), it was estimated that a further 0·032 QALYs would be gained by the REACT intervention versus control treatment (in addition to the 0·040 QALYs estimated in the trial). These QALY gains come solely from health-related quality of life as it relates to SPPB—a woman aged 75 years receiving the REACT programme would have an estimated 0·317 additional life-years spent with an SPPB score between 8 and 12 and 0·317 fewer life-years spent with an SPPB score below 8. It was also estimated that cost savings would grow over the lifetime of the recipient, from £103 within the first 24 months to £290, again due to spending more time with a more favourable SPPB score. This result means that, after extrapolating long-term costs and outcomes, the REACT programme would still dominate the control treatment (table 3).

**Table 3:** Cost-effectiveness results from the decision analytic model

		**Costs**	**QALYs**
**Deterministic analyses**			
Woman aged 75 years			
	Intervention group	£20 338	7·183
	Control group	£20 627	7·111
	Difference	−£290	0·072
Man aged 65 years			
	Intervention group	£23 583	9·726
	Control group	£23 783	9·669
	Difference	−£200	0·058
**Probabilistic sensitivity analyses**			
Woman aged 75 years			
	Intervention group	£22 655	6·865
	Control group	£22 999	6·785
	Difference (95% credible interval)	−£343 (−£376 to −£311)	0·081 (0·078 to 0·083)
Man aged 65 years			
	Intervention group	£25 460	9·441
	Control group	£25 677	9·376
	Difference (95% credible interval)	−£218 (−£248 to −£187)	0·064 (0·063 to 0·066)

QALY=quality-adjusted life-year.

**Figure 3:**
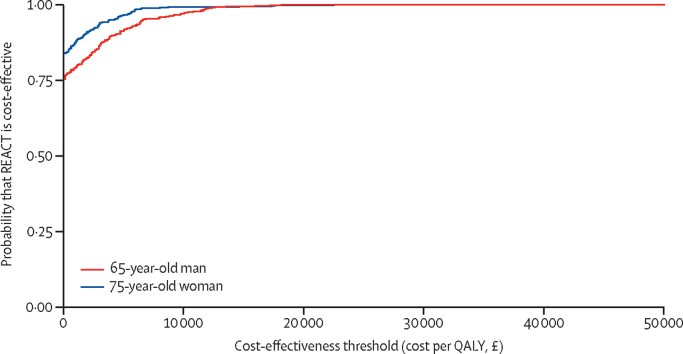
Cost-effectiveness acceptability curves from model-based economic evaluation QALY=quality-adjusted life-year.

When the age and sex of the recipient was varied, we established that the REACT programme would be least cost-effective for men aged 65 years, although it would still dominate the control treatment. The REACT programme would be most cost-effective for women aged 85 years. When an age and sex composition representative of the current UK general population aged 65–100 years was used, the REACT programme was expected to result in cost savings of £273 and 0·068 incremental QALYs compared with the control treatment. Probabilistic sensitivity analysis was also done (figure 3), as were multiple one-way sensitivity analyses for model parameters (appendix 1 p 10).

## Discussion

This analysis suggests that the REACT programme is cost-effective in comparison to usual care, generating net cost savings and improved health-related quality of life. The cost of delivering the REACT programme was estimated to be £622 per participant, which could be more than offset by reductions in NHS and PSS resource use, in particular relating to secondary care. Even if it were conservatively assumed that such reductions in secondary care use would not be realised in a real-life setting, the REACT programme would still be considered cost-effective as the short-term gain of 0·040 QALYs has a monetary value of £800–1200 (if QALYs are valued at £20 000–30 000 per QALY). Extrapolation beyond the trial duration of 24 months suggests further savings and QALY gains would be realised, because the difference in mobility between those receiving the REACT programme and control participants is expected to persist for some time and lead to improved quality of life and lower use of NHS and PSS resources. The economic analyses assume no cost sharing with participants—ie, the full cost of the intervention is borne by the NHS and PSS. It is possible that commissioners might prefer to use a cost-sharing approach—our analysis suggests this is unnecessary for the programme to be cost-effective, and our study cannot advise on how a cost to participants might affect uptake or affect socioeconomic inequality.

These economic analyses have strengthened the previous finding that lower extremity function (measured specifically with SPPB) is associated with health-care resource utilisation,^2^ and have also estimated the association between SPPB and preference-based quality of life.

There are some limitations with the economic data collected within the trial. We estimated resource use through the use of questionnaires with 6-month recall administered at baseline and at 6, 12, and 24 months after randomisation. These data are at risk of recall bias, and resource use was not measured for a 6-month period (12–18 months after randomisation), but was imputed from resource use in the preceding and following 6-month periods. Resource use questionnaires were felt to be the most appropriate way to capture resource utilisation because the population was at risk of making considerable use of social care and informal care, for which administrative data are unavailable.

There was a moderate amount of missing economic data, as only 451 (58%) of 777 participants were complete cases for the economic evaluation. Participants in the intervention group who attended a greater proportion of sessions were also more likely to be complete cases. Both the base-case analysis (using multiple imputation) and the complete-case analysis found the REACT programme to be cost-effective, but there remains a risk that data were missing not at random, which could lead to some bias in both analyses.

The modelling approach adopted to extrapolate long-term costs and QALYs to a lifetime horizon (as is generally necessary for economic evaluations in the UK) was conservative. We assumed no difference in the ability to sustain physical function between the groups, despite the incorporation of behaviour change components in the intervention. No assumptions were made regarding the possible beneficial effect of increased physical activity on circulatory diseases and other chronic conditions. We did not include any causal effect of mobility and mortality, although there is considerable observational data that an association exists.^4^ As a result of these conservative assumptions, it is possible that we have underestimated the total benefits of the REACT programme.

A limitation of our modelling analysis is that it relied on estimating the associations between SPPB and costs and utilities from REACT trial participants, who, due to selection criteria for the trial, entered the trial with fewer comorbidities than average and with an initial SPPB in the range 4 to 9 (so estimates of resource use and quality of life outside this range only arise in follow-up data). Further research will be required to validate the costs and health-state utility values in representative samples, particularly in those with poor lower extremity function.

The cost of delivering the intervention is also somewhat uncertain and is expected to be subject to market forces. For example, the intervention delivery costs are sensitive to the costs of REACT session leaders' time and venue hire, both of which will be sensitive to local and regional variations in supply and demand of qualified labour and suitable venues. We suggest that local commissioners should generate local delivery cost estimates to ensure value for money is obtained without dissuading potential providers.

The REACT programme was designed as an adaptation of the Lifestyle Interventions and Independence for Elders (LIFE) intervention^17^ so that it could be delivered as a cost-effective community-based programme. The REACT programme has shown a similar gain in QALYs as the LIFE study over the trial period: 0·040 QALYs over 2 years versus 0·047 QALYs over 2·6 years.^18^

The REACT programme has been shown to be effective^5^ and cost-effective for maintaining mobility in retirement-age adults at risk of major mobility-related disability. The REACT programme has not been evaluated for individuals outside this group (eg, with no current mobility limitations, or with severe limitations) so its cost-effectiveness in such individuals is unknown.

## Data sharing

Data and analysis scripts will be made available on request by qualified scientific and medical researchers for legitimate research purposes. Collected patient-level data will be deidentified and will include at least all variables necessary to reproduce the analyses. The health economic analysis plan will be made available on request. Requests should be sent to the corresponding author. Data will be available on request for 6 months from the date of publication. Investigators are invited to submit study proposal requests detailing research questions and hypotheses to receive access to these data.

## Declaration of interests

CG was a committee member of the National Institute for Health Research (NIHR) General Funding Committee during 2019–20. AM-L is a member of the NIHR Health Technology Assessment Committee, the South West for Research for Patient Benefit Programme, the NIHR Global Health Units Research Funding Committee, and the NIHR Global Health Groups Research Funding Committee. All other authors declare no competing interests.

## References

[1] Groessl EJ, Kaplan RM, Rejeski WJ (2019). Physical activity and performance impact long-term quality of life in older adults at risk for major mobility disability. Am J Prev Med.

[2] Stathi A, Withall J, Greaves CJ (2018). A community-based physical activity intervention to prevent mobility-related disability for retired older people (REtirement in ACTion (REACT)): study protocol for a randomised controlled trial. Trials.

[3] Simmonds B, Fox K, Davis M (2014). Objectively assessed physical activity and subsequent health service use of UK adults aged 70 and over: a four to five year follow up study. PLoS One.

[4] Pavasini R, Guralnik J, Brown JC (2016). Short Physical Performance Battery and all-cause mortality: systematic review and meta-analysis. BMC Med.

[5] Stathi A, Greaves CJ, Thompson JL (2022). Effect of a physical activity and behaviour maintenance programme on functional mobility decline in older adults: the REACT (Retirement in Action) randomised controlled trial. Lancet Public Health.

[6] National Institute for Health and Care Excellence (April 4, 2013). Guide to the methods of technology appraisal 2013: the reference case. https://www.nice.org.uk/process/pmg9/chapter/the-reference-case.

[7] Husereau D, Drummond M, Petrou S (2013). Consolidated Health Economic Evaluation Reporting Standards (CHEERS) statement. BMJ.

[8] Withall J, Greaves CJ, Thompson JL (2020). The tribulations of trials: lessons learnt recruiting 777 older adults into REtirement in ACTion (REACT), a trial of a community, group-based active ageing intervention targeting mobility disability. J Gerontol A Biol Sci Med Sci.

[9] Herdman M, Gudex C, Lloyd A (2011). Development and preliminary testing of the new five-level version of EQ-5D (EQ-5D-5L). Qual Life Res.

[10] Ware JE, Sherbourne CD (1992). The MOS 36-item short-form health survey (SF-36). Conceptual framework and item selection. Med Care.

[11] van Hout B, Janssen MF, Feng YS (2012). Interim scoring for the EQ-5D-5L: mapping the EQ-5D-5L to EQ-5D-3L value sets. Value Health.

[12] Kharroubi SA, Brazier JE, Roberts J, O'Hagan A (2007). Modelling SF-6D health state preference data using a nonparametric Bayesian method. J Health Econ.

[13] Brazier J, Ratcliffe J, Salomon J, Tsuchiya A (2007).

[14] Curtis L, Burns A (2018).

[15] Improvement NHS (2020). National Cost Collection for the NHS. https://www.england.nhs.uk/national-cost-collection/.

[16] Ciani O, Buyse M, Drummond M, Rasi G, Saad ED, Taylor RS (2017). Time to review the role of surrogate end points in health policy: state of the art and the way forward. Value Health.

[17] Pahor M, Guralnik JM, Ambrosius WT (2014). Effect of structured physical activity on prevention of major mobility disability in older adults: the LIFE study randomized clinical trial. JAMA.

[18] Groessl EJ, Kaplan RM, Castro Sweet CM (2016). Cost-effectiveness of the LIFE physical activity intervention for older adults at increased risk for mobility disability. J Gerontol A Biol Sci Med Sci.

