# Supplementary material for: Cost-effectiveness of a physical activity and behaviour maintenance programme on functional mobility decline in older adults: an economic evaluation of the REACT (Retirement in Action) trial
Source: Lancet Public Health. 2022 Mar 21;7(4):e327–34. doi: 10.1016/S2468-2667(22)00030-5 (PMC8967720; doi:10.1016/S2468-2667(22)00030-5)
Supplement: Supplementary appendix 1 [file mmc1.pdf]

THE LANCET
Public Health

Supplementary appendix 1

This appendix formed part of the original submission and has been peer reviewed. We post it as supplied by the authors.

Supplement to: Snowsill TM, Stathi A, Green C, et al. Cost-effectiveness of a physical activity and behaviour maintenance programme on functional mobility decline in older adults: an economic evaluation of the REACT (Retirement in Action) trial. *Lancet Public Health* 2022; published online March 21. https://doi.org/10.1016/S2468-2667(22)00030-5.

APPENDIX

Item	Unit cost	Source
**Primary and/or community-based services (per contact or consultation)**		
GP at surgery/health centre	39·19	PSSRU 2019[1] (General practitioner cost per surgery consultation lasting 9·22 minutes, including direct care staff costs, including training)
GP via telephone	15·52	PSSRU 2019[1]
GP home visit	78·92	PSSRU 2014[3] and PSSRU 2019[1] (£156 per hour of GMS activity, 11·4 minutes visit, 1:0·61 direct-to-indirect time ratio (7·0 minutes indirect), 12 minutes travel)
Practice nurse at surgery/health centre	12·43	PSSRU 2014[3] and PSSRU 2019[1] (£37 per hour, 15.5 minutes per contact, 1:0.30 direct-to-indirect time ratio)
Practice nurse via telephone	7·80	PSSRU 2019[1]
Practice nurse at home	39·68	National Cost Collection 2018/19[2] (N02AF – District Nurse, Face to face)
Physiotherapist	62·90	National Cost Collection 2018/19[2] (A08A1 – Physiotherapist, Adult, One to one)
Occupational therapist	83·17	National Cost Collection 2018/19[2] (A06A1 – Occupational Therapist, Adult, One to one)
Nutritionist	89·90	National Cost Collection 2018/19[2] (A03 – Dietician)
Chiropodist	42·51	National Cost Collection 2018/19[2] (A09A – Podiatrist, Tier 1, General Podiatry)
Counsellor	45·00	PSSRU 2019[1] (Agenda for Change band 6 for 1 hour)
Walk-in-Centre	21·00	PSSRU 2019[1] (Assume 15 minutes of Band 6 nurse time)
**Overnight hospital stays (per attendance)**		
General medical ward or long-stay ward	385·16 plus 503·96 per day	NHS Reference Costs 2017/18[4] inflated to 2018/19 prices[1]
Intensive care unit	3,532·09	National Cost Collection 2018/19[2]
Other hospital usage		
Outpatient appointment	126·85	National Cost Collection 2018/19[2] (Weighted average of all Consultant-Led and Non-Consultant-Led Outpatient attendances)
Day case treatment	751·90	National Cost Collection 2018/19[2] (Weighted average of all Day Case episodes)
A&E attendance	166·05	National Cost Collection 2018/19[2] (Weighted average of all Accident & Emergency episodes)
**Social care services [a]**		
Day care centre (per day)	40·20	PSSRU 2019[1]
Convalescent or nursing home	79·73	PSSRU 2019[1]
Paid care at home (per hour)	18·95	PSSRU 2019[1]

Supplementary table 1: Unit costs of NHS and PSS resources

Note: [a] It is estimated that 33% of long-term care spending comes from voluntary and out-of-pocket expenditure,[5] and this is excluded from the unit costs in the NHS and PSS perspective

1. Curtis L, Burns A. *Unit Costs of Health and Social Care 2019*. Canterbury: Personal Social Services Research Unit, University of Kent; 2018. http://dx.doi.org/10.22024/UniKent/01.02.79286

2. NHS Improvement. *National Cost Collection for the NHS*. 2020. URL: https://improvement.nhs.uk/resources/national-cost-collection/ (Accessed February 20, 2020).

3. Curtis L. *Unit Costs of Health and Social Care 2014*. Canterbury: Personal Social Services Research Unit, University of Kent; 2014.

4. NHS Improvement. *Reference costs*. 2018. URL: https://improvement.nhs.uk/resources/reference-costs/

5. Incisive Health. *An international comparison of long-term care funding and outcomes: Insights for the social care green paper*. 2018. URL: https://www.incisivehealth.com/wp-content/uploads/2018/08/international_comparison_of_social_care_funding_and_outcomes.pdf (Accessed 8 June, 2020).

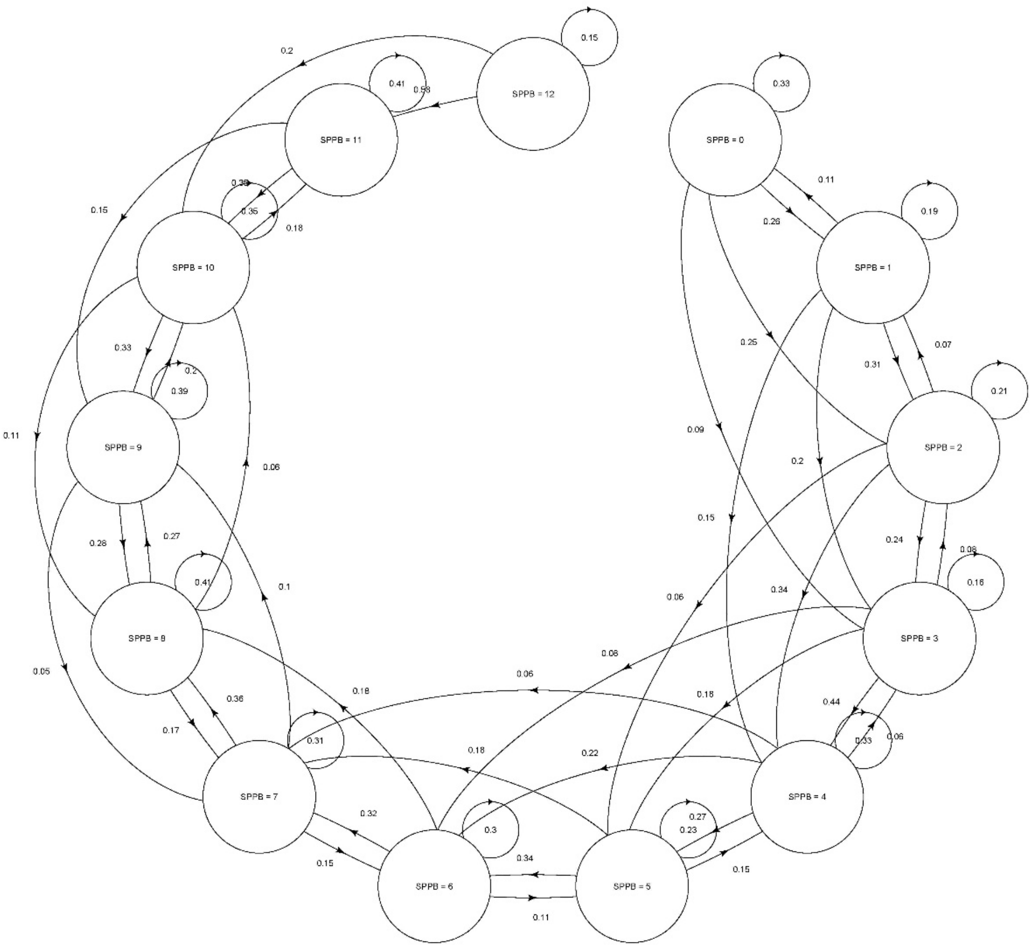

Supplementary figure 1: Diagram of the Markov model for estimating long-term costs and outcomes

Notes: For clarity, the Dead state is omitted and only transition probabilities ≥ 0.05 are shown. Transition probabilities have been calculated for the third cycle (between 24 and 36 months post-randomisation) for a 75-year-old woman.)

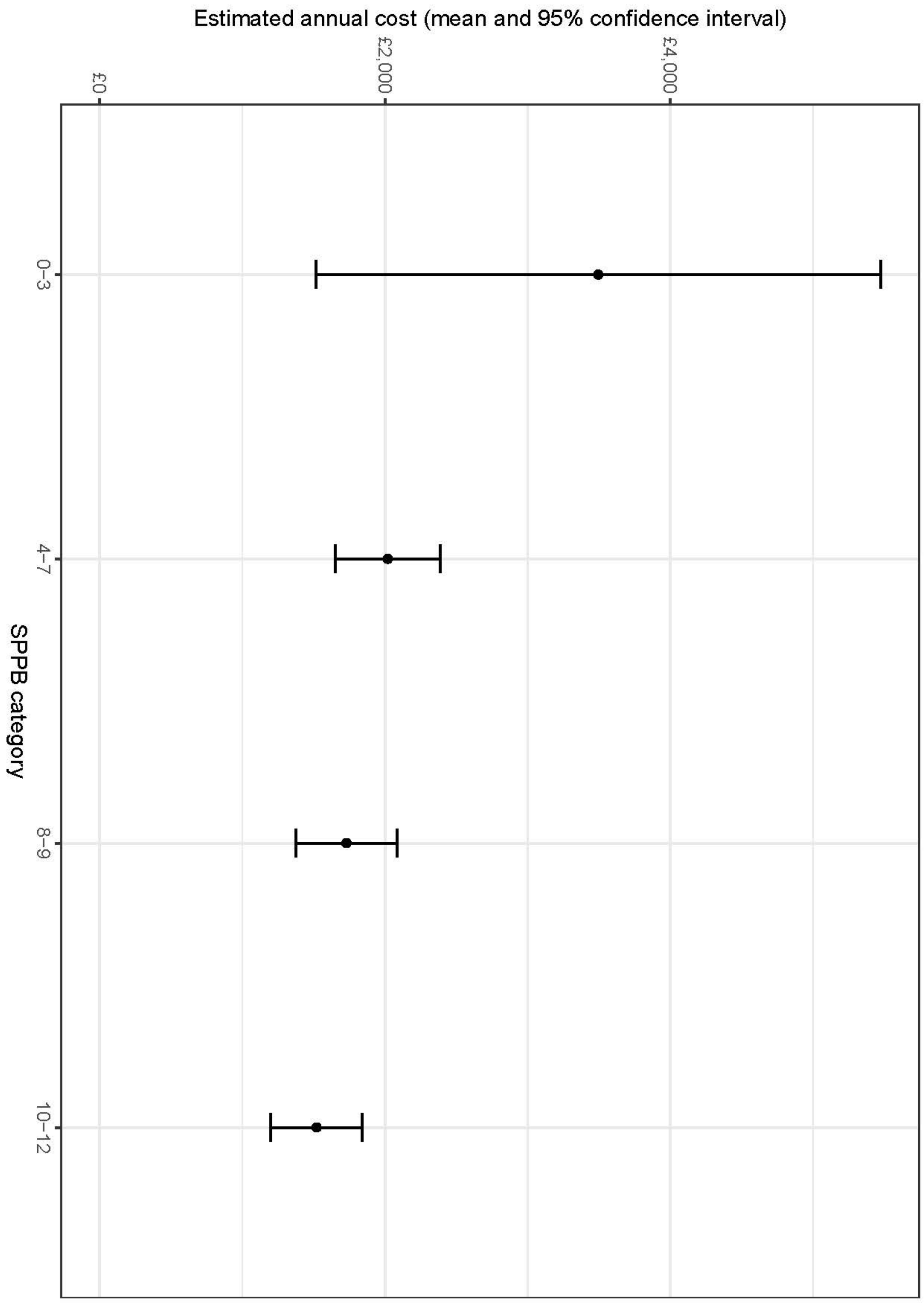

Supplementary figure 2: Cost by SPPB range

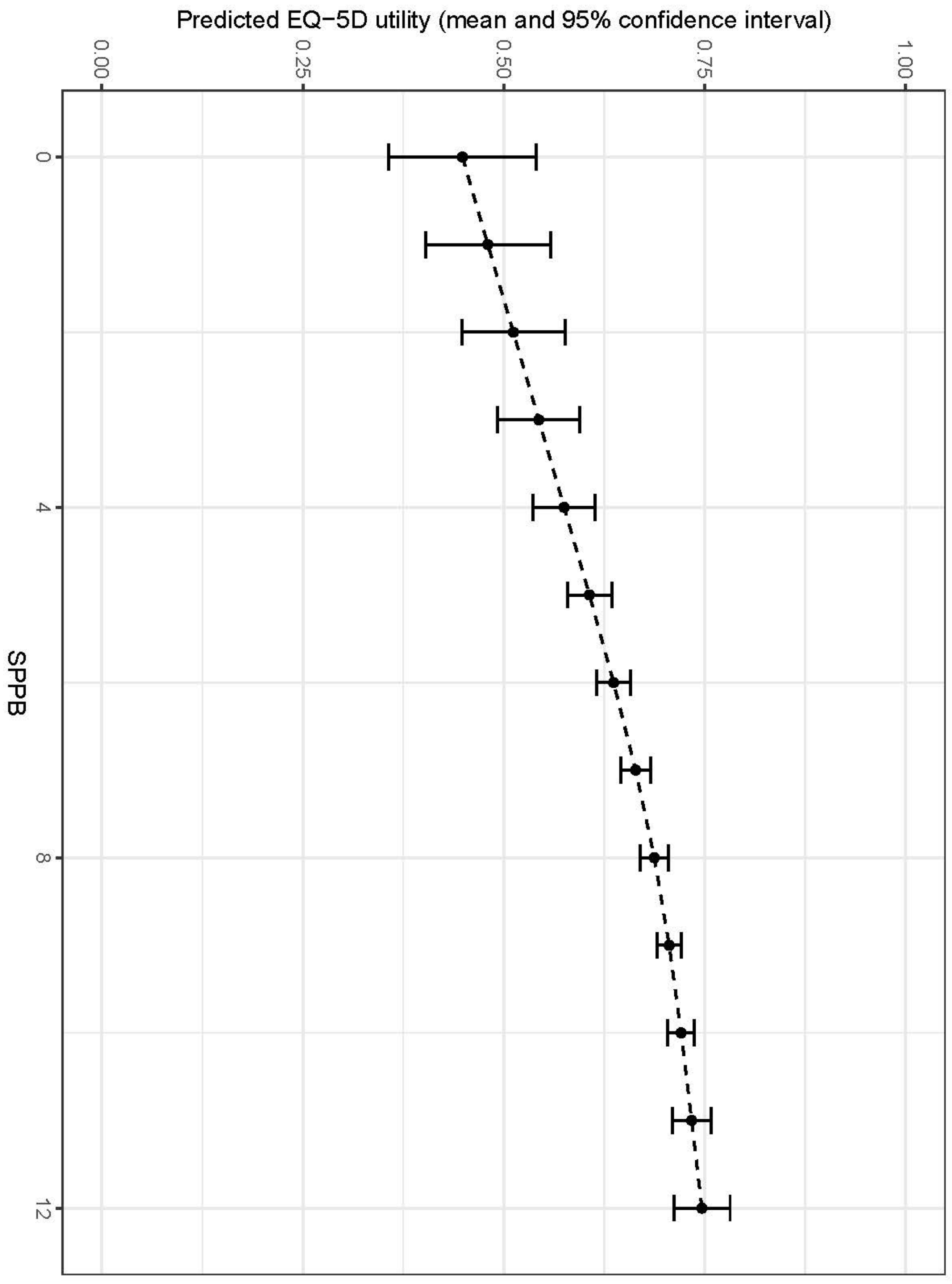

Supplementary figure 3: Predicted EQ-5D utility according to SPPB. *Note: A restricted cubic regression spline with knots at SPPB = 4, SPPB = 8 and SPPB = 11 was used. Further details are given in Supplementary File 1.*

HRQoL	REACT				Control			
	Baseline	6 months	12 months	24 months	Baseline	6 months	12 months	24 months
EQ-5D-5L dimensions, n (%)								
Mobility	400	346	339	330	358	299	294	303
1	91 (23%)	122 (35%)	108 (32%)	102 (31%)	78 (22%)	70 (23%)	71 (24%)	66 (22%)
2	154 (38%)	104 (30%)	109 (32%)	89 (27%)	120 (34%)	98 (33%)	98 (33%)	90 (30%)
3	124 (31%)	95 (17%)	95 (28%)	103 (31%)	135 (38%)	100 (33%)	100 (34%)	109 (36%)
4	30 (8%)	25 (7%)	27 (8%)	33 (10%)	24 (7%)	30 (10%)	23 (8%)	35 (12%)
5	1 (0%)	0 (0%)	0 (0%)	3 (1%)	1 (0%)	1 (0%)	2 (1%)	3 (1%)
Self-care	400	346	338	330	359	299	294	303
1	321 (80%)	287 (83%)	273 (81%)	254 (77%)	278 (77%)	227 (76%)	235 (80%)	237 (78%)
2	53 (13%)	43 (12%)	53 (16%)	59 (18%)	58 (16%)	57 (19%)	44 (15%)	46 (15%)
3	24 (6%)	15 (4%)	11 (3%)	14 (4%)	20 (6%)	13 (4%)	15 (5%)	15 (5%)
4	1 (0%)	1 (0%)	1 (0%)	1 (0%)	3 (1%)	2 (1%)	0 (0%)	2 (1%)
5	1 (0%)	0 (0%)	0 (0%)	2 (1%)	0 (0%)	0 (0%)	0 (0%)	3 (1%)
Usual activities	399	346	339	330	356	299	294	303
1	148 (37%)	171 (49%)	150 (44%)	134 (41%)	146 (41%)	114 (38%)	118 (40%)	113 (37%)
2	153 (38%)	105 (30%)	119 (34%)	112 (34%)	127 (36%)	115 (38%)	96 (33%)	114 (38%)
3	77 (19%)	61 (18%)	120 (19%)	64 (19%)	69 (19%)	57 (19%)	64 (22%)	59 (19%)
4	16 (4%)	8 (2%)	11 (3%)	14 (4%)	12 (3%)	10 (3%)	12 (4%)	8 (3%)
5	5 (1%)	1 (0%)	3 (1%)	6 (2%)	2 (1%)	3 (1%)	4 (1%)	9 (3%)
Pain / discomfort	400	346	339	330	358	300	294	303
1	53 (13%)	51 (15%)	56 (17%)	52 (16%)	53 (15%)	39 (13%)	37 (13%)	36 (12%)
2	169 (42%)	157 (45%)	156 (46%)	139 (42%)	140 (39%)	121 (40%)	134 (46%)	118 (39%)
3	146 (36%)	105 (30%)	94 (28%)	104 (32%)	124 (35%)	103 (34%)	95 (32%)	109 (36%)

HRQoL	REACT				Control			
	**Baseline**	**6 months**	**12 months**	**24 months**	**Baseline**	**6 months**	**12 months**	**24 months**
4	29 (7%)	31 (9%)	30 (9%)	31 (9%)	36 (10%)	32 (11%)	22 (7%)	32 (11%)
5	3 (1%)	2 (1%)	3 (1%)	4 (1%)	5 (1%)	5 (2%)	6 (2%)	8 (3%)
Anxiety / depression	397	346	338	330	357	300	293	302
1	249 (63%)	219 (63%)	223 (66%)	209 (63%)	220 (62%)	190 (63%)	189 (65%)	196 (65%)
2	106 (27%)	96 (28%)	86 (25%)	97 (29%)	97 (27%)	74 (25%)	69 (24%)	76 (25%)
3	40 (10%)	25 (7%)	25 (7%)	21 (6%)	34 (10%)	27 (9%)	32 (11%)	29 (10%)
4	2 (0%)	5 (1%)	3 (1%)	2 (1%)	6 (2%)	8 (3%)	2 (1%)	1 (0%)
5	0 (0%)	1 (0%)	1 (0%)	1 (0%)	0 (0%)	1 (0%)	1 (0%)	0 (0%)
EQ-5D value, Mean (n, SD)								
Crosswalk to EQ-5D-3L	0.689 (397, 0.158)	0.708 (346, 0.167)	0.705 (337, 0.170)	0.686 (330, 0.200)	0.677 (352, 0.165)	0.670 (299, 0.181)	0.680 (293, 0.183)	0.661 (302, 0.661)
EQ-5D-VAS	70.6 (399, 17.3)	72.6 (349, 16.7)	71.4 (338, 17.8)	70.4 (329, 18.4)	72.1 (362, 16.9)	72.0 (298, 17.3)	70.6 (294, 17.0)	69.4 (301, 19.2)
EQ-5D-5L value set	0.789 (397, 0.149)	0.805 (346, 0.160)	0.801 (337, 0.158)	0.782 (330, 0.180)	0.781 (352, 0.152)	0.770 (299, 0.177)	0.785 (293, 0.162)	0.767 (302, 0.174)
SF-36, Mean (n, SD)								
PCS	29.7 (393, 11.0)	32.8 (342, 11.5)	31.9 (334, 11.5)	30.9 (326, 12.0)	30.0 (352, 10.6)	30.6 (293, 10.9)	29.8 (293, 10.9)	29.2 (295, 10.8)
MCS	54.6 (393, 8.3)	54.4 (342, 8.6)	54.0 (334, 8.8)	54.3 (326, 8.6)	53.8 (352, 8.7)	54.0 (293, 9.1)	54.3 (293, 9.4)	54.5 (295, 9.1)
SF-6D	0.622 (365, 0.095)	0.637 (323, 0.103)	0.637 (313, 0.098)	0.630 (312, 0.105)	0.623 (326, 0.089)	0.621 (280, 0.103)	0.622 (280, 0.100)	0.619 (283, 0.096)

Supplementary table 2: Health-related quality of life measured in the REACT study

HRQoL	REACT				Control			
	**Baseline**	**6 months**	**12 months**	**24 months**	**Baseline**	**6 months**	**12 months**	**24 months**
EQ-5D-5L dimensions, n (%)								
Mobility	400	346	339	330	358	299	294	303
1	91 (23%)	122 (35%)	108 (32%)	102 (31%)	78 (22%)	70 (23%)	71 (24%)	66 (22%)
2	154 (38%)	104 (30%)	109 (32%)	89 (27%)	120 (34%)	98 (33%)	98 (33%)	90 (30%)
3	124 (31%)	95 (17%)	95 (28%)	103 (31%)	135 (38%)	100 (33%)	100 (34%)	109 (36%)
4	30 (8%)	25 (7%)	27 (8%)	33 (10%)	24 (7%)	30 (10%)	23 (8%)	35 (12%)
5	1 (0%)	0 (0%)	0 (0%)	3 (1%)	1 (0%)	1 (0%)	2 (1%)	3 (1%)
Self-care	400	346	338	330	359	299	294	303
1	321 (80%)	287 (83%)	273 (81%)	254 (77%)	278 (77%)	227 (76%)	235 (80%)	237 (78%)
2	53 (13%)	43 (12%)	53 (16%)	59 (18%)	58 (16%)	57 (19%)	44 (15%)	46 (15%)
3	24 (6%)	15 (4%)	11 (3%)	14 (4%)	20 (6%)	13 (4%)	15 (5%)	15 (5%)
4	1 (0%)	1 (0%)	1 (0%)	1 (0%)	3 (1%)	2 (1%)	0 (0%)	2 (1%)
5	1 (0%)	0 (0%)	0 (0%)	2 (1%)	0 (0%)	0 (0%)	0 (0%)	3 (1%)
Usual activities	399	346	339	330	356	299	294	303
1	148 (37%)	171 (49%)	150 (44%)	134 (41%)	146 (41%)	114 (38%)	118 (40%)	113 (37%)
2	153 (38%)	105 (30%)	119 (34%)	112 (34%)	127 (36%)	115 (38%)	96 (33%)	114 (38%)
3	77 (19%)	61 (18%)	120 (19%)	64 (19%)	69 (19%)	57 (19%)	64 (22%)	59 (19%)
4	16 (4%)	8 (2%)	11 (3%)	14 (4%)	12 (3%)	10 (3%)	12 (4%)	8 (3%)
5	5 (1%)	1 (0%)	3 (1%)	6 (2%)	2 (1%)	3 (1%)	4 (1%)	9 (3%)
Pain / discomfort	400	346	339	330	358	300	294	303
1	53 (13%)	51 (15%)	56 (17%)	52 (16%)	53 (15%)	39 (13%)	37 (13%)	36 (12%)
2	169 (42%)	157 (45%)	156 (46%)	139 (42%)	140 (39%)	121 (40%)	134 (46%)	118 (39%)
3	146 (36%)	105 (30%)	94 (28%)	104 (32%)	124 (35%)	103 (34%)	95 (32%)	109 (36%)

HRQoL	REACT				Control			
	Baseline	6 months	12 months	24 months	Baseline	6 months	12 months	24 months
4	29 (7%)	31 (9%)	30 (9%)	31 (9%)	36 (10%)	32 (11%)	22 (7%)	32 (11%)
5	3 (1%)	2 (1%)	3 (1%)	4 (1%)	5 (1%)	5 (2%)	6 (2%)	8 (3%)
Anxiety / depression	397	346	338	330	357	300	293	302
1	249 (63%)	219 (63%)	223 (66%)	209 (63%)	220 (62%)	190 (63%)	189 (65%)	196 (65%)
2	106 (27%)	96 (28%)	86 (25%)	97 (29%)	97 (27%)	74 (25%)	69 (24%)	76 (25%)
3	40 (10%)	25 (7%)	25 (7%)	21 (6%)	34 (10%)	27 (9%)	32 (11%)	29 (10%)
4	2 (0%)	5 (1%)	3 (1%)	2 (1%)	6 (2%)	8 (3%)	2 (1%)	1 (0%)
5	0 (0%)	1 (0%)	1 (0%)	1 (0%)	0 (0%)	1 (0%)	1 (0%)	0 (0%)
EQ-5D value, Mean (n, SD)								
Crosswalk to EQ-5D-3L	0.689 (397, 0.158)	0.708 (346, 0.167)	0.705 (337, 0.170)	0.686 (330, 0.200)	0.677 (352, 0.165)	0.670 (299, 0.181)	0.680 (293, 0.183)	0.661 (302, 0.661)
EQ-5D-VAS	70.6 (399, 17.3)	72.6 (349, 16.7)	71.4 (338, 17.8)	70.4 (329, 18.4)	72.1 (362, 16.9)	72.0 (298, 17.3)	70.6 (294, 17.0)	69.4 (301, 19.2)
EQ-5D-5L value set	0.789 (397, 0.149)	0.805 (346, 0.160)	0.801 (337, 0.158)	0.782 (330, 0.180)	0.781 (352, 0.152)	0.770 (299, 0.177)	0.785 (293, 0.162)	0.767 (302, 0.174)
SF-36, Mean (n, SD)								
PCS	29.7 (393, 11.0)	32.8 (342, 11.5)	31.9 (334, 11.5)	30.9 (326, 12.0)	30.0 (352, 10.6)	30.6 (293, 10.9)	29.8 (293, 10.9)	29.2 (295, 10.8)
MCS	54.6 (393, 8.3)	54.4 (342, 8.6)	54.0 (334, 8.8)	54.3 (326, 8.6)	53.8 (352, 8.7)	54.0 (293, 9.1)	54.3 (293, 9.4)	54.5 (295, 9.1)
SF-6D	0.622 (365, 0.095)	0.637 (323, 0.103)	0.637 (313, 0.098)	0.630 (312, 0.105)	0.623 (326, 0.089)	0.621 (280, 0.103)	0.622 (280, 0.100)	0.619 (283, 0.096)

Supplementary table 2: Health-related quality of life measured in the REACT study

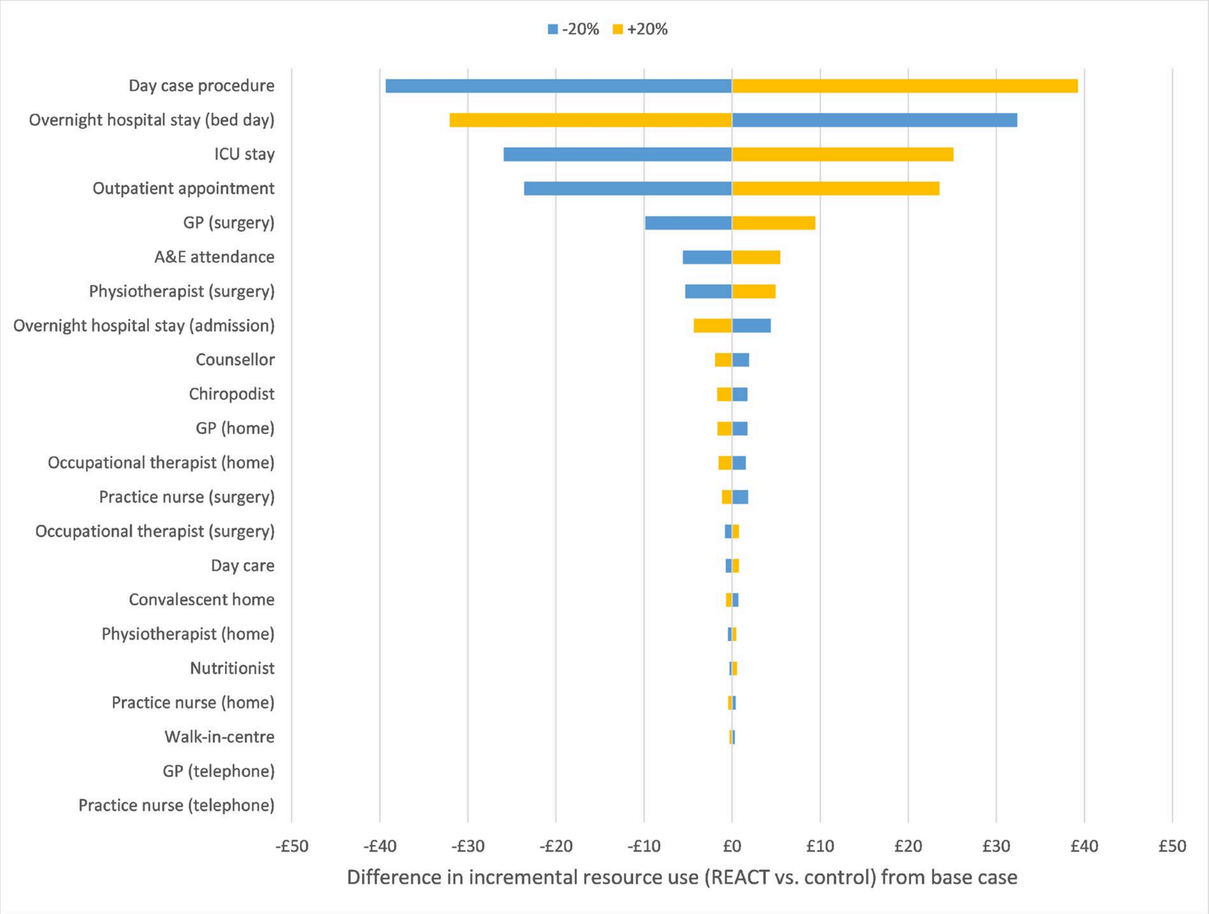

Supplementary figure 4: Comparison of resource use between REACT and control participants

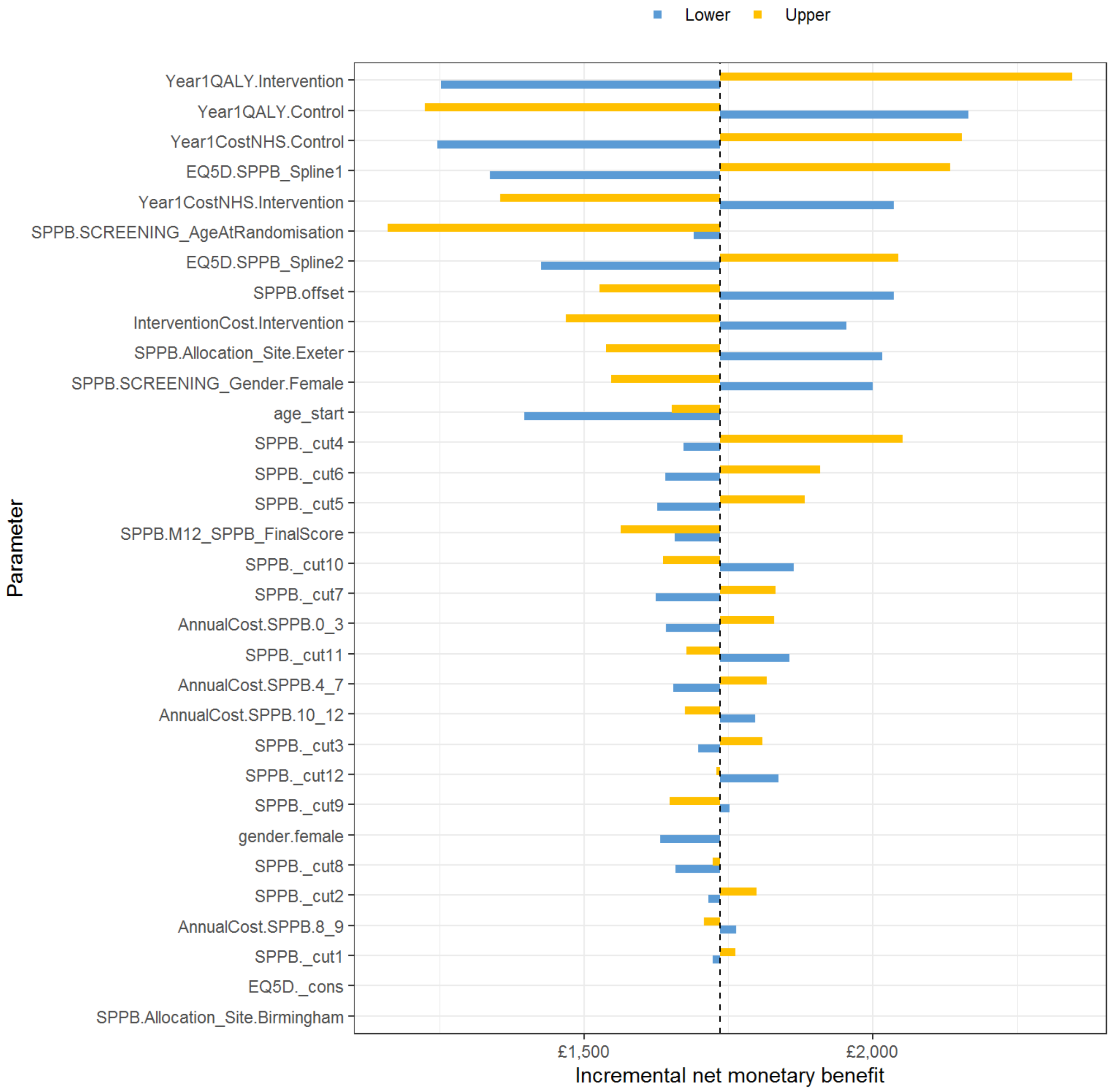

Supplementary figure 5: Sensitivity analyses for model parameters
